# Biological Pretreatment of Rubberwood with *Ceriporiopsis subvermispora* for Enzymatic Hydrolysis and Bioethanol Production

**DOI:** 10.1155/2013/268349

**Published:** 2013-09-19

**Authors:** Forough Nazarpour, Dzulkefly Kuang Abdullah, Norhafizah Abdullah, Nazila Motedayen, Reza Zamiri

**Affiliations:** ^1^Institute of Bioscience, Universiti Putra Malaysia, 43400 Serdang, Selangor, Malaysia; ^2^Department of Chemistry, Faculty of Science, Universiti Putra Malaysia, 43400 Serdang, Selangor, Malaysia; ^3^Department of Chemical and Environmental Engineering, Faculty of Engineering, Universiti Putra Malaysia, 43400 Serdang, Selangor, Malaysia; ^4^Department of Materials Engineering and Ceramic, CICECO, University of Aveiro, Campus Santiago, 3810-193 Aveiro, Portugal

## Abstract

Rubberwood (*Hevea brasiliensis*), a potential raw material for bioethanol production due to its high cellulose content, was used as a novel feedstock for enzymatic hydrolysis and bioethanol production using biological pretreatment. To improve ethanol production, rubberwood was pretreated with white rot fungus *Ceriporiopsis subvermispora* to increase fermentation efficiency. The effects of particle size of rubberwood (1 mm, 0.5 mm, and 0.25 mm) and pretreatment time on the biological pretreatment were first determined by chemical analysis and X-ray diffraction and their best condition obtained with 1 mm particle size and 90 days pretreatment. Further morphological study on rubberwood with 1 mm particle size pretreated by fungus was performed by FT-IR spectra analysis and SEM observation and the result indicated the ability of this fungus for pretreatment. A study on enzymatic hydrolysis resulted in an increased sugar yield of 27.67% as compared with untreated rubberwood (2.88%). The maximum ethanol concentration and yield were 17.9 g/L and 53% yield, respectively, after 120 hours. The results obtained demonstrate that rubberwood pretreated by *C. subvermispora* can be used as an alternative material for the enzymatic hydrolysis and bioethanol production.

## 1. Introduction

In recent years, rising gas prices and environmental concerns cause the driving force for developing alternative energy sources, especially fuel ethanol for automobiles [1]. Unlike fossil fuel, ethanol has the advantages of being renewable, cleaner having a burning, and producing no greenhouse gases [2]. Lignocellulosic biomass is an attractive option for the sustainable production of fuels [3]. Therefore, using lignocellulosic biomass as the feedstock reduces the costs of bioethanol production as a result of its widespread availability, sustainable production, and cheap availability [4]. Currently, rubber tree (*Hevea brasiliensis*) which is also known as *Hevea* wood is the most abundant lignocellulosic material in Malaysia which can be used as a potential raw material for bioethanol production due to its high cellulose content (53.01%) [5].

Lignocellulosic materials are composed mainly of cellulose, hemicellulose, and lignin. Pretreatment, as the first step towards conversion of lignocellulose to ethanol, removes lignin and hemicellulose, reduces cellulose crystallinity, and increases the porosity of materials [6]. In general, pretreatment can be classified into physical pretreatment physicochemical pretreatment, chemical pretreatment, and biological pretreatment [7]. However, physical and chemical pretreatment need high temperature treatment with acid or alkali, which disrupts lignocellulose and also results in acidic or caustic hydrolysate and the production of inhibitory byproducts [8, 9]; therefore, such pretreatment methods increase the cost of processes with neutralization or washing step that results in the loss of sugars [6, 10]. Consequently, it is necessary to develop a benign alternative to harsh chemicals.

Biological pretreatment applies microorganisms especially fungi and their enzyme systems to delignify cellulosic feedstocks [11]. White rot fungi with high selectivity of lignin degradation over cellulose are important for successful microbial pretreatment. Most white rot fungi, such as *Phanerochaete chrysosporium*, simultaneously degrade holocellulose (cellulose and hemicellulose) and lignin, resulting in a low cellulose recovery [12, 13]. Some species preferentially degrade lignin and part of the hemicellulose, leaving a cellulose rich residue [13, 14]. *Ceriporiopsis subvermispora* is one of the most investigated white rot fungi for pretreatment because of its selectively for lignin biodegradation with very low cellulose loss [15]. 

In this research, we used a woody biomass (rubberwood) as an alternative material for the fungal pretreatment and bioethanol production. To date no such work has been reported. The objective of this study was to evaluate the effects of fungal pretreatment of rubberwood by *C. subvermispora* on enzymatic hydrolysis and ethanol production. The changes in lignin, cellulose, and hemicellulose after pretreatment, hydrolysis conversion efficiency, and ethanol yield from fermentation were measured to evaluate pretreatment effectiveness. The effects of particle size on degradation of rubberwood are also investigated.

## 2. Materials and Methods

### 2.1. Microorganism

*Ceriporiopsis subvermispora* FP-90031-Sp (ATCC 90467) was purchased from American Type Culture Collection (ATCC) and maintained as a frozen culture (−80°C) in 30% glycerol. The fungus was incubated on 2.4% potato dextrose agar (PDA) plates at 28°C for 7 days. 

### 2.2. Biomass Preparation

The wood was chipped using the lab scale chipper. The chips then were transferred to a Pallman disc flaker and cut to smaller particle size. After flaking, they were ground to pass through 0.25, 0.5, and 1.00 mm screens, respectively, and approximately dried to 5% moisture content in an oven at 103 ± 2°C for 24 h.

### 2.3. Fungal Pretreatment with *C. subvermispora*

Seven grams of rubberwood with 0.25, 0.5, and 1 mm particle sizes was mixed with 12 mL distilled water to obtain an optimal moisture content of 75% [12], and this mixture was autoclaved at 121°C for 20 min and then cooled and aseptically inoculated with a plug (9 mm in diameter) from the plate culture to obtain the effect of fungus on the rubberwood. Parafilm was wrapped around flasks to act as a barrier against moisture loss and contamination. Small perforations were made to the film to avoid moisture condensation and allow ventilation of chambers. Fungal pretreatment was carried out in an incubator at 28°C under static conditions for 30, 60, and 90 days. A set of nonpretreated sterilized woods were used as controls. After pretreatment, the flasks were stored at 4°C for compositional analysis, enzymatic hydrolysis, and ethanol production. All tests were performed in triplicate [16].

### 2.4. Enzymatic Hydrolysis

Enzymatic hydrolysis was conducted following NREL laboratory analytical procedure LAP-008 [17]. Cellulase (Celluclast 1.5 L, produced by *Trichoderma reesei*) with activity of 70 FPU/mL, supplemented with *β*-glucosidase (Novozyme 188, produced by *Aspergillus niger*) with activity of 122 CBU/mL, was used. Enzymatic hydrolysis experiments were carried out in 250 mL bottles. Each bottle was loaded with 1% w/w effective cellulose content, 1% w/v yeast extract, 2% w/v peptone, and 0.05 M citrate buffer (pH 4.8) in a final working weight of 50 grams and autoclaved at 121°C for 15 min. After cooling, cellulase (25 FPU/g cellulose) was added and supplemented with *β*-glucosidase (60 CBU/g cellulose) to avoid inhibition due to cellobiose accumulation [5]. After hydrolyzing at 50°C for 168 h in an incubator shaker set at 150, hydrolysis was terminated by boiling in a water bath for exactly 5 min to inactivate cellulase before being chilled on ice. Hydrolyzed samples were centrifuged at 10,000 rpm for 5 min. The supernatants were recovered for reducing sugars analysis at least in triplicate.

### 2.5. Production of Bioethanol

Production of bioethanol was carried out by simultaneous saccharification and fermentation (SSF) following NREL LAP-008 procedure. The microorganism used for the fermentation was *Saccharomyces cerevisiae* D5A purchased from ATCC, USA. 

The strain was maintained in glycerol vials at −80°C for use as a working stock. It was cultured on YPD plates containing yeast extract 10 g/L, peptone 20 g/L, dextrose 20 g/L, and agar 20 g/L. Plates were incubated at 37°C for 2 days. A loopful of yeast was taken from a single colony of YPD plate and used to inoculate 100 mL of YPD media containing 10 g/L yeast extract, 20 g/L peptone, and 50 g/L dextrose [18] in a 250 mL flask. The media were sterilized by filtration and the flasks were autoclaved without media at 121°C [19]. The flask was incubated for 10–14 h in an incubator shaker operating at 37°C and 130 rpm. The cells were harvested, washed with DI water twice by centrifugation (5000 rpm for 5 min), and used for inoculating SSF flasks [20]. SSF was then carried out in a 250 mL serum bottle containing 6% w/w cellulose, 1% w/v yeast extract, 2% w/v peptone, and 0.05 M citrate buffer (pH 4.8) in a final working weight of 100 grams. Bottles were sparged with nitrogen, sealed, and autoclaved at 121°C for 15 min. After cooling, medium was inoculated with *S. cerevisiae* D5A (10% v/v, starting OD. 0.5) and cellulase enzyme was injected at a dose of 25 FPU/g cellulose and supplemented with *β*-glucosidase at a dose of 60 CBU/g cellulose to avoid inhibition due to cellobiose accumulation [5]. The SSF was conducted at 37°C and 130 rpm for 7 days. Samples were taken after 0, 3, 24, 48, 72, 96, 120, 144, and 168 h, filtered, and stored at −20°C for ethanol and reducing sugar analysis. All assays were performed at least in triplicate. The ethanol produced was analyzed qualitatively using gas chromatography. The ethanol yield (%) was calculated using the following equation:

(1) ethanol  yield  (%) =g  of  ethanol  in  culture  broth  ×100g  of  glucan  in  culture  broth  ×1.1  ×0.511.

### 2.6. Analysis Methods

#### 2.6.1. Subexperiment 1: Analysis of Chemical Composition

The total solids content (also called the percent dry weight) was determined according to the Laboratory Analytical Procedure No. 001 (LAP-001) from the National Renewable Energy Laboratory (NREL) [21]. The extractives were removed from analyzed samples by Soxhlet extraction with acetone (6 h), according to the procedure adapted from TAPPI standard T 204 om-97. The percentage of acid-insoluble lignin was determined according to TAPPI procedure (T224 om-88). The holocellulose content was determined according to DIN 2403. The *α*-cellulose content of rubber was determined according to TAPPI 203 om-93. The percentage of hemicellulose was calculated by subtracting the percent *α*-cellulose from holocellulose. Dry mass loss was calculated as the percentage of total solids loss after pretreatment. Lignin degradation, cellulose loss, and hemicellulose loss were defined as the percentages of lignin, cellulose, and hemicellulose reduction.

#### 2.6.2. Subexperiment 2: Determination of Reducing Sugar

Total reducing sugar was determined by the 3, 5-dinitrosalicylic acid (DNS) method using glucose as the standard [22]. The samples were analyzed using a spectrophotometer (Shimadzu, Columbia, MD, USA) at 540 nm. The absorbance readings were then converted into equivalent sugar concentration (mg/mL) using a standard glucose solution curve. Reducing sugar yield was calculated using the following equation:

(2) reducing  sugar  yield  (%) =reducing  sugar  produced  ×0.9  ×100amount  of  H  rubberwood,

where H are cellulose and hemicellulose.

#### 2.6.3. Subexperiment 3: X-Ray Diffraction Analysis

The X-ray diffractograms were obtained with a Rigaku Geigeflex Diffractometer with Cu and K*α* radiation at 30 kV and 30 mA. The diffraction intensity was measured between Bragg angles (2*θ*) of 5°−50° at the speed of 2°/min. The crystallinity index (CrI) was calculated using the intensities of crystalline region at 2*θ* = 22.5° and amorphous region 2*θ* = 18°, respectively, using the following equation [23]:

(3) $$\begin{array}{c} \text{crystallinity}\;\text{index}\;\left(\text{CrI}\right)\%=\frac{I_{\text{crystalline}}-I_{\text{amorphous}}}{I_{\text{crystalline}}}, \end{array}$$

where *I*_crystalline_ is the intensity of crystalline region and *I*_amorphous_ is the intensity of amorphous region.

#### 2.6.4. Subexperiment 4: Fourier Transform Infrared Spectroscopy (FT-IR) Analysis

The FT-IR spectra of untreated and treated rubberwood were obtained by direct transmittance using the KBr pellet technique [24]. Spectra were recorded with a Perkin Elmer 1650 FT-IR spectrometer (Perkin Elmer, Waltham, MA, USA). The spectra (4000–500 cm^−1^) were measured at a spectral resolution of 4 cm^−1^ and 64 scans per sample.

#### 2.6.5. Subexperiment 5: Scanning Electron Microscopy (SEM)

The morphologies of untreated and pretreated rubberwood were examined using scanning electron microscopy (SEM). Before analysis, samples were mounted on metal stubs by double tape and the surface was coated with gold to avoid charging. Images were taken at 15 kV by PHILLIPS XL30 ESEM.

#### 2.6.6. Subexperiment 6: Gas Chromatography

The ethanol produced from the fermentation process was analyzed qualitatively after distillation. The qualitative analysis was carried out using gas chromatography Shimadzu GC-14B with a BP21 capillary column (30 m × 0.250 mm ID, 0.25 *µ*m film) and GC-flame ionization detector (GC-FID). Initial and final oven temperatures were 40 and 130°C, detector temperature was 250°C, and injector temperature was 230°C. 

#### 2.6.7. Subexperiment 7: Statistical Analysis

All experiments (chemical compositions and sugar yield) were carried out in triplicate and the values are an average of the three values obtained within a 95% confidence level. The effects of biological pretreatment on lignin, hemicellulose, and cellulose reduction during biological pretreatments were analyzed using the Statistical Analysis Software (SAS) program (released 6.12, 1988.SAS Institute Inc., Cary, NC).

## 3. Results and Discussion

### 3.1. Effect of Particle Size on Degradation of Rubberwood by *C. subvermispora*

#### 3.1.1. Analysis of Chemical Composition

Particle size of the substrate is important. It affects the performance of solid state fermentation [20]. Generally, reduced particle size provides larger surface area for microbial attack but leads to limitation in interparticle space availability and heat transfer. Compared to smaller particle size, bigger particles provide better aeration/respiration opportunities but result in lesser surface area. Hence, determination of particle size corresponding to optimum growth and enzyme production is necessary [25]. The effects of particle size (1 mm, 0.5 mm, and 0.250 mm) on degradation of rubberwood by *C. subvermispora* during 90 days are shown in Table 1. As shown in Table 1, higher lignin degradation was obtained with 1 mm rubberwood (16.65%) than was obtained with 0.5 and 0.250 mm rubberwood (15.13% and 13.10%, resp.) after 30 days. Moreover, weight losses of hemicellulose were 25.13%, 19.75%, and 17.80%, respectively, with 1, 0.5, and 0.250 mm. However, there was no significant difference between the cellulose degradation of all particle sizes (*P* > 0.05). 

For rubberwood with the particle sizes of 0.5 and 0.250 mm, substantially lower lignin degradation (29.52% and 29.65%, resp.) was observed compared to 1 mm particle size of rubberwood (37.30%) after 60 days. The hemicellulose degradation for 1 mm rubberwood particle size is 36.02% which is higher than 0.5 mm particle size, while no significant difference was seen in hemicellulose degradation for 0.250 mm with 1 mm and 0.5 mm. The lowest cellulose caused in 1 mm particle size of rubberwood.

Fungal pretreated rubberwood with all particle sizes lost significant (*P* < 0.05) lignin compared to untreated rubberwood after 90 days. The weight losses of lignin for 1.00, 0.50, and 0.25 mm were 45.06%, 38.78%, and 34.89%, respectively. Higher hemicellulose reduction was obtained with 1 mm particle size (42.08%) compared to 0.50 and 0.25 mm particle sizes (39.91% and 38.76%, resp.). The cellulose reduction obtained with 1 mm particle size (10.5%) was lower than those particle sizes.

Selectivity value, the lignin/cellulose loss ratio, was used to evaluate the selective lignin-degrading ability [26]. The selectivity values increased with increasing pretreatment time because *C. subvermispora* can effectively reduce recalcitrance of rubberwood with high selectivity of lignin degradation rate and minimal cellulose loss. The highest selectivity value of 4.29% with lignin degradation of 45.06% was obtained from 1 mm particle size of rubberwood after 90 days.

#### 3.1.2. X-Ray Diffraction

X-ray diffraction is the best option to estimate the impacts of pretreatment on crystalline regions of cellulose. The crystallinity index (CrI), which is a measure of the amount of crystalline cellulose, was calculated according to Segal's empirical method [22]. The crystallinity data of untreated and fungal treated rubberwood at different particle sizes are shown in Table 2. The results show that there is a significant increase in the crystallinity between the pretreated and untreated wood. The CrI values of 1 mm, 0.5 mm, and 0.250 mm samples increased 23.59%, 19.20%, and 17.64%, respectively, compared with untreated wood during 90 days of pretreatment. The increase in the crystallinity is expected due to the degradation and modification of amorphous components as reported in previous studies [27, 28]. The amorphous part may include amorphous cellulose, hemicellulose, and lignin [28, 29]. Therefore, the CrI increase in pretreated samples indicates that the amorphous portion of the rubberwood was more removed than the crystalline portion and cellulose became more exposed after pretreatment. According to Table 1, the higher lignin and hemicellulose reduction and lower cellulose loss were obtained with 1 mm particle size compared to other particle sizes. The crystallinity data also indicated that samples with 1 mm particle size had higher CrI value which is consistent with the obtained results shown in Table 1. Based on the chemical analysis and X-ray diffraction results, we may conclude that 1 mm is the best considered size for the biological pretreatment. Hence, rubberwood with 1 mm particle size was chosen for the rest of the study.

### 3.2. FT-IR Analysis

#### 3.2.1. Undecayed Rubberwood

**Figure 1:**
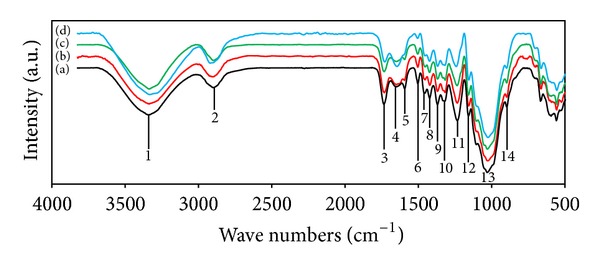
FT-IR spectra of rubberwood samples at various degrees of decay by *C*. *subvermispora*: (a) undecayed wood, (b) decayed for 30 d, (c) decayed for 60 d, and (d) decayed for 90 d.

FT-IR spectroscopy was used to demonstrate the physical structures and functional groups of the lignocellulosic materials. FT-IR spectroscopy of undecayed rubberwood is shown in Figure 1. The absorbance peaks in the 3400–3300 cm^−1^  (1) region were attributed to the stretching of O–H groups, whereas those around 2900–2800 cm^−1^  (2) were due to the stretching of C–H [30]. The peak located at 1735 cm^−1^  (3) was assigned to the C=O stretching of the acetyl group in hemicellulose [31]. The peaks in the region between 1620 and 1650 cm^−1^ (4 and 5) for all samples were characterized by the absorbed water [32]. The absorbance at 1504 cm^−1^ (6) is attributed to aromatic skeletal vibrations in lignin [31]. The peaks located at 1428 and 1458 cm^−1^ (7 and 8) were assigned to the C–H deformation in lignin and carbohydrates [33]. The peaks observed in the range 1380–1320 cm^−1^ (9 and 10) in all samples were attributed to the bending vibration of C–H and C–O groups of the aromatic ring in polysaccharides [32]. The absorption located at 1234 cm^−1^ (11) is caused by O–H phenolic in lignin [34]. The absorbances at 1158 and 898 (12 and 14) cm^−1^ correspond to C–O–C vibration in cellulose and hemicellulose, and C–H deformation in cellulose, respectively [31]. The C–O–C pyranose ring skeletal vibrations occur in the region 1102 to 1024 cm^−1^ (13) [35]. The peaks below 898 cm^−1^ are of little importance in the characterization of cellulose.

#### 3.2.2. Wood Decayed by *C. subvermispora*

FT-IR spectra of rubberwood (1 mm) exposed to *C. subvermispora* for 30, 60, and 90 days are shown in Figure 1. The intensities of carbohydrate bands at 1369 and 898 cm^−1^ were slightly decreased. The constant intensity of the carbohydrate band at 1158 cm^−1^ is remarkable. In particular, the effect of fungal attack on the wood is clearly noticeable by increasing intensity of the 1647 cm^−1^ band (conjugated carbonyl groups, mainly originating from lignin) and the significant decreasing intensities at 1593, 1504, and 1234 cm^−1^ with exposure time [36]. However, a significant decrease in the intensity of carbonyl absorption band at 1735 cm^−1^ also indicated the decay of xylan (hemicellulose) by *C. subvermispora*. From the FT-IR spectra analysis, it could be concluded that *C. subvermispora* improved the degradation of lignin but it should have little effect on degradation of carbohydrates which result from a selective lignin removal. These findings are in agreement with Ferraz et al. [37]. 

### 3.3. Scanning Electron Microscopy (SEM)

**Figure 2:**
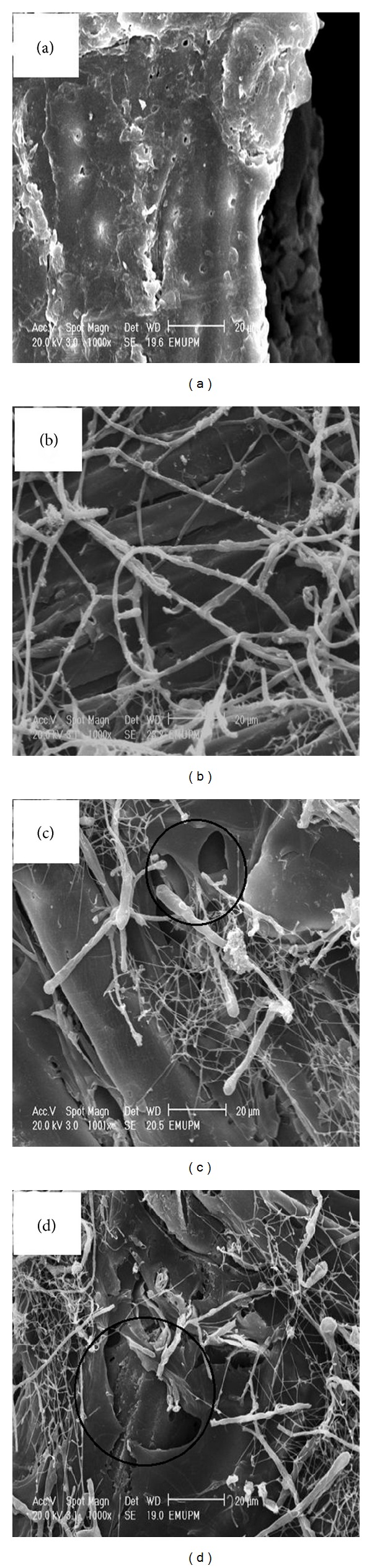
Scanning electron microscopy images of rubberwood pretreated with *C. subvermispora*. (a) Untreated rubberwood; (b) rubberwood pretreated with *C. subvermispora* for 30 d, (c) 60 d, and (d) 90 d.

**Figure 3:**
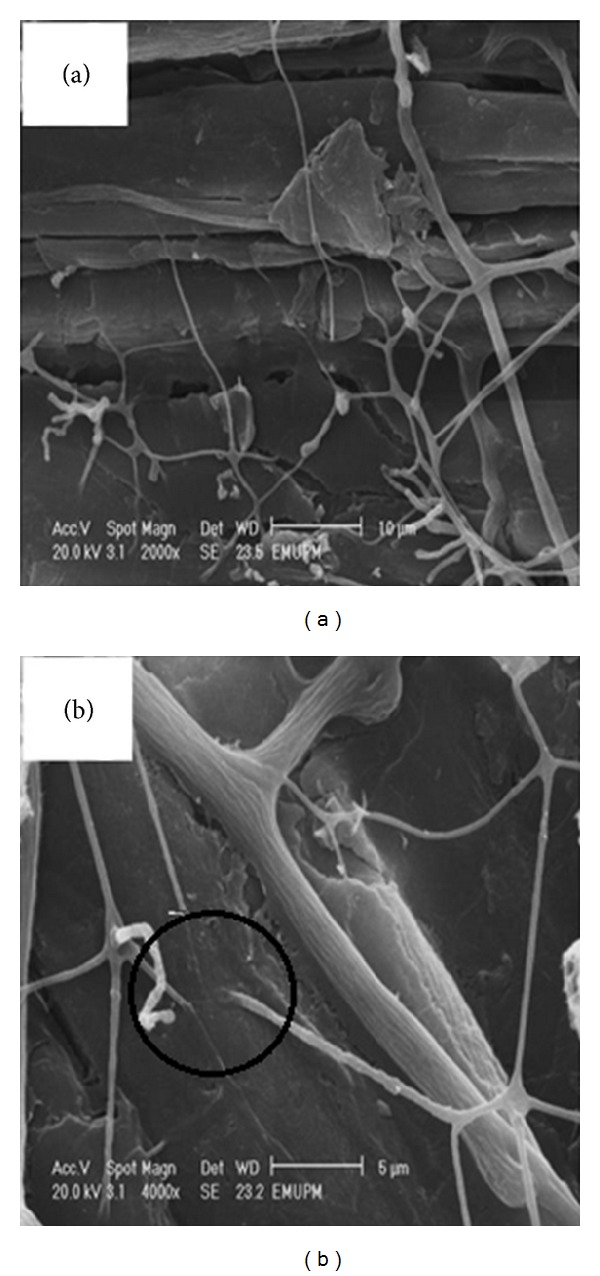
SEM images of pretreated rubberwood (a) showing hyphae penetration through cell walls; (b) same image at higher magnification.

The morphology of the untreated and pretreated rubberwood with 1 mm particle size by *C. subvermispora* was investigated using scanning electron microscopy (SEM) for 30, 60, and 90 days (Figures 2(a), 2(b), 2(c), and 2(d)). SEM images showed that lignocellulose in the untreated rubberwood had an intact surface structure (Figure 3(a)), while the pretreated rubberwood had a rugged and partially broken face which resulted from the removal of lignin and breaking of lignocelluloses networks during the pretreatment (marked circle in Figures 2(c) and 2(d)). As shown in Figure 2(b), rubberwood was quickly colonized by the fungus with the formation of abundant white mycelial mass on the wood chips after 30 days of pretreatment. The mycelial mass increased with biodegradation time, becoming whitish after 90 days of pretreatment (Figure 2(d)). SEM images also showed that in all treated samples, branched hyphae covered the surface of the wood chips. Mycelial mass increased during degradation. Hyphae penetrate the chips through the lumen and pit fiber wood cells (Figures 3(a) and 3(b)). 

### 3.4. Enzymatic Hydrolysis

**Figure 4:**
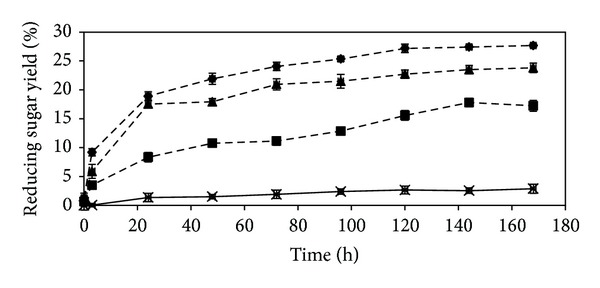
Time course of reducing sugar yield (%) during the hydrolysis of rubberwood. (×) untreated; (- - -) pretreated with *C. subvermispora*, for (■) 30, (▲) 60, and (♦) 90 days. Error bars represent standard error.

Lignin plays an important role in biomass recalcitrance to cellulolytic enzymes. It is generally well recognized that low lignin content results in high cellulose digestibility.  Figure 4 shows the time courses of the production of reducing sugars using the untreated and fungal pretreated rubberwood for 0, 3, 24, 48, 72, 96, 120, 144, and 168 h as we have already published in our previous work [38]. As expected, the higher rate of enzymatic hydrolysis exhibited by pretreated samples was due to removal of the physical protective coat of cellulose and consequently the improved cellulose digestibility. Compared to pretreated samples, rubberwood without pretreatment was much more resistant to enzymatic hydrolysis, producing only 2.88% fermentable sugar yield after 168 h of hydrolysis. The reducing sugar yield from sample treated for 30 days was 17.23%. The reducing sugar yields increased with the cultivation time beyond 30 days, reaching 23.80% for samples pretreated for 60 days. Further increase was observed when the cultivation time was further extended to 90 days. The highest reducing sugar yield reached about 27.67% for samples pretreated for 90 days. These results show that the enzymatic hydrolysis yield of rubberwood is considerably affected by the cultivation time and the reducing sugar yield heavily depends on the extent of delignification and hemicellulose removal from the lignocellulosic materials [39]. This explains why sample pretreated by *C. subvermispora* resulted in high sugar yield (27.67%). It was reported that after 120 days of cultivation by a newly isolated fungus, *Echinodontium taxodii* 2538 on two native woods: *Chinese willow* (hard wood) and *China-fair* (soft wood), the enzymatic hydrolysis yield showed a significant increase (4.7-fold for hard wood and 3-fold for soft wood) [26]. By contrast, pretreatment of rubberwood (hard wood) by *C. subvermispora* resulted in much higher enzymatic hydrolysis yields (9.6-fold) during a relatively short degradation period. Lee et al. [40] reported lower sugar yields (21.01%, 14.91%, and 15.03%) from soft wood *Pinus densiflora* pretreated with *Stereum hirsutum*, *Polyporus brumalis*, and *Ceriporia lacerate*, respectively, compared to rubberwood treated with *C. subvermispora* for 72 h at 50°C. However, the sugar yield obtained from wood treated with *C. subvermispora* (230.6 mg sugar/g rubberwood) in this study is comparable with that obtained by Zhang et al. (232.2 mg sugar/g bamboo) but in much shortened pretreatment time (90 days compared to 60–120 days by Zhang et al.) [41].

### 3.5. Production of Bioethanol

**Figure 5:**
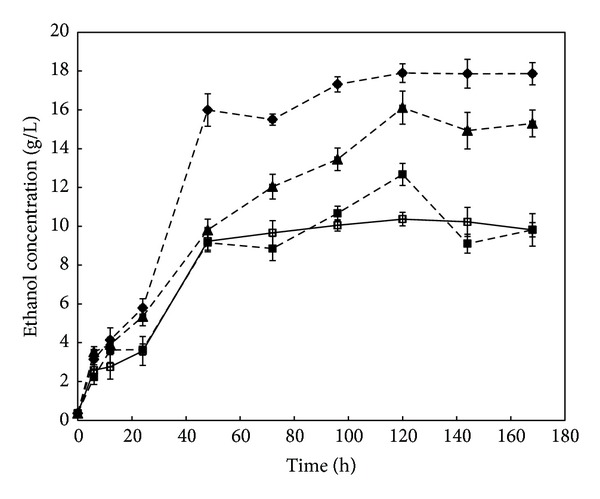
Time course of bioethanol concentration (g/L) of untreated and pretreated rubberwood by *C. subvermispora*. (□) untreated and (■) pretreated rubberwood after 30, (▲) 60, and (♦) 90 days. Error bars represent standard error.

Bioethanol production was carried out by simultaneous saccharification and fermentation (SSF) process. The rubberwood samples pretreated with *C. subvermispora* for 30, 60, and 90 days were used in the bioethanol production. Untreated sample was used as control. The bioethanol produced by the fermentation process increased within the first 48 h of SSF and maximum value was obtained after 120 h (Table 3 and Figure 5). After 120 h, the maximum bioethanol concentration and yield were 17.9 g/L and 53% yield, respectively. For fungal pretreated rubberwood for 30 and 60 days, the ethanol concentrations obtained were 12.7 g/L and 16.1 g/L, respectively. The bioethanol concentration and yield of untreated sample were 10.4 g/L and 30.7% after 120 h fermentation. 

These results are comparable to that obtained from pretreated corn stover with *C. subvermispora,* where an overall ethanol yield of 57.80% was achieved [20]. Another report showed that a combination of *C. subvermispora* and ethanolysis pretreatment resulted in an overall ethanol yield of 62% [15]. This higher yield is expected due to additional ethanolysis treatment compared to this study using only *C. subvermispora* pretreatment. Bak et al. [9] reported that when fungal fermented rice straw with *Dichomitus squalens* was used as a substrate in SSF, the bioethanol yield was 54.2% after 24 h. Other works on production of ethanol from biomass treated with microorganism were 0.017 g of ethanol/g of corn stover after 144 h fermentation [42] and 0.004–0.027 g of ethanol/g of dry lignocelluloses after 48 h fermentation [43]. The bioethanol yields from these previously conducted studies are much lower than the yield obtained in this study (0.003–0.143 g of ethanol/g of dry rubberwood). The results of this work show that fungal pretreatment of rubberwood increased the enzymatic digestibility of cellulose that led to high bioethanol yield.

## 4. Conclusions

We may conclude that fungal pretreatment using* C. subvermispora* provides a cost-effective method for reducing the recalcitrance of rubberwood with high selectivity of lignin degradation rate and minimal cellulose loss for enzymatic hydrolysis and bioethanol production. The study showed that lignin degradation rate was significantly affected by the particle size of rubberwood and time of the pretreatment. XRD and chemical analysis of control and pretreated rubberwood with different particle sizes showed that rubberwood with the particle size of 1 mm was efficiently degraded due to providing better aeration/respiration opportunities compared to smaller particle sizes. FT-IR spectra analysis and SEM observations of control and pretreated rubberwood with 1 mm particle size showed equivalent results with chemical analysis. The results of this work also revealed that rubberwood with high cellulose content can be successfully converted to bioethanol by the SSF process.

## Figures and Tables

**Table 1:** Degradation of rubberwood pretreated by *C. subvermispora* at different particle sizes for 30, 60, and 90 days^1^.

Particle size (mm)	Selectivity value^2^	Weight loss (%)		
		Lignin	Hemicellulose	Cellulose
0.25				
30	2.16^b^	13.10^c^ (0.36)	17.80^c^ (0.67)	6.06^a^ (0.75)
60	2.46^b^	29.65^b^ (0.31)	34.35^a,b^ (0.36)	12.07^a^ (0.29)
90	2.75^c^	34.89^c^ (0.9)	38.76^b^ (0.88)	12.69^a^ (0.83)

0.50				
30	2.78^a,b^	15.13^b^ (0.19)	19.75^b^ (0.27)	5.44^a^ (0.50)
60	2.75^b^	29.52^b^ (0.23)	34.01^b^ (0.46)	10.73^a,b^ (0.92)
90	3.21^b^	38.78^b^ (0.27)	39.91^b^ (0.44)	12.08^a^ (0.37)

1.00				
30	3.57^a^	16.65^a^ (1.08)	25.13^a^ (0.69)	4.66^a^ (1.26)
60	3.95^a^	37.30^a^ (1.31)	36.02^a^ (1.48)	9.44^b^ (1.42)
90	4.29^a^	45.06^a^ (0.82)	42.08^a^ (1.16)	10.50^b^ (0.98)

^1^Standard deviations of three replicates in parentheses; letters on the right side of the data in the same column indicated significant levels (*P* < 0.05 ANOVA, *F*(3, 6));^2^ selectivity value = lignin loss/cellulose loss.

**Table 2:** Crystallinity index of untreated and treated rubberwood by *C. subvermispora* at different particle sizes for 30, 60, and 90 days.

Samples	CrI (%)		
	30 d	60 d	90 d
Untreated	43.12	43.12	43.12
0.25	52.66	58.25	60.77
0.50	47.66	59.68	62.32
1.00	52.37	65.84	66.71

**Table 3:** Bioethanol yields (%) of untreated and pretreated rubberwood by *C. subvermispora* after 30, 60, and 90 days (samples 1, 2, and 3, resp.)^1^.

Time (h)	Control	Sample 1	Sample 2	Sample 3
24	10.6 (1.2)	10.8 (2.4)	15.8 (0.9)	17.2 (0.6)
48	27.4 (0.8)	27.1 (1.9)	29.1 (1.3)	47.4 (1.4)
72	28.7 (2.1)	26.3 (1.7)	35.7 (2.5)	46.06 (2.3)
96	29.8 (1.7)	31.6 (0.7)	39.9 (3.1)	51.4 (2.8)
120	30.7 (2.3)	37.6 (1.5)	47.8 (2.2)	53.1 (3.5)
144	30.3 (1.4)	27.0 (2.6)	44.3 (3.7)	53.0 (2.9)

^1^Standard deviations of three replicates are in parentheses.
